# Nanowire-Based Electrode for Acute *In Vivo* Neural Recordings in the Brain

**DOI:** 10.1371/journal.pone.0056673

**Published:** 2013-02-19

**Authors:** Dmitry B. Suyatin, Lars Wallman, Jonas Thelin, Christelle N. Prinz, Henrik Jörntell, Lars Samuelson, Lars Montelius, Jens Schouenborg

**Affiliations:** 1 Division of Solid State Physics and The Nanometer Structure Consortium, Lund University, Lund, Sweden; 2 Neuronano Research Center, Medical Faculty, Lund University, Lund, Sweden; 3 Department of Measurement Technology and Industrial Electrical Engineering, Lund University, Lund, Sweden; Faculty of Medicine University of Leipzig, Germany

## Abstract

We present an electrode, based on structurally controlled nanowires, as a first step towards developing a useful nanostructured device for neurophysiological measurements *in vivo*. The sensing part of the electrode is made of a metal film deposited on top of an array of epitaxially grown gallium phosphide nanowires. We achieved the first functional testing of the nanowire-based electrode by performing acute *in vivo* recordings in the rat cerebral cortex and withstanding multiple brain implantations. Due to the controllable geometry of the nanowires, this type of electrode can be used as a model system for further analysis of the functional properties of nanostructured neuronal interfaces *in vivo*.

## Introduction

Implantable neural interfaces [1]–[3] have the potential to revolutionize neuroscience research and clinical therapy [4]–[6], but still suffer from a number of shortcomings related to *e.g.* instability with respect to recorded neurons and tissue reactions that encapsulate and insulate the implant [7]. Since the recording properties depend, to a large extent, on the electrode surface properties and the tissue reactions to the surface, research on nanostructured surfaces in order to improve recording properties of neural interfaces is crucial. Indeed, nanostructured electrodes are considered as a promising alternative to conventional neuronal interfaces [8]–[11] since they may provide advantages such as a better spatial resolution, a shorter cell-to-electrode distance [12]–[14], as well as improved electrical properties [12], [15]–[18]. They also have a potential for better biocompatibility [18]–[21], less tissue damage [12], [13], [18], [22] and new functionalities, such as selective guidance of neuronal fibers [23]. Importantly, cell signal recordings with different nanowire-based electrodes have recently been achieved *in vitro* [8]–[10], [12]–[14], [24], [25] and it has been shown that the small diameter of epitaxially grown wires may provide a minimally invasive tissue penetration [12]–[14], [26], [27]. However, *in vivo* studies of nanostructured neuronal electrodes have, so far, only been performed using carbon nanotubes without structural features control and in combination with rather big surfaces [15], [17]. Hence, further research on nanostructured neural interfaces with structural features control is needed in order to interface the nervous tissue in an optimal way and ultimately allow constructions of electrodes for *in vivo* neuronal recordings on the sub cellular level with minimal side effects.

Nanowires are high aspect ratio nanostructures, which have attracted a lot of attention lately due to their applications in different fields ranging from efficient energy harvesting to biological applications [28]–[32]. Recently, it has been shown that epitaxially grown gallium phosphide (GaP) nanowires have beneficial properties for neuronal interfaces such as improved cell survival [21] and improved cell adhesion with focal adhesions forming specifically on the nanowires [33]. GaP nanowires can be synthesized with a high aspect ratio (>50), very little tapering and exceptional control over their position and geometry, compared to other material nanowires [34].

Here we report the design and fabrication of a first generation of GaP nanowire-based electrode with a controllable nanomorphology. We achieved the first functional testing of the device *in vivo* by performing acute recordings in the rat cerebral cortex. The nanowires were used as a backbone for metal nanostructured electrode with a three-dimensional (3D) structure. With this electrode design we provide a first step of the development of a new model system for further research on the functionality of nanostructure-based neuronal interfaces *in vivo*, with the prospect of enabling a more intimate contact between the electrode and the neurons, as well as a more reliable tissue anchoring [35], thus providing a better electrode-cell electrical coupling [14], [25], [36].

## Results

### Structure and Materials

**Figure 1:**
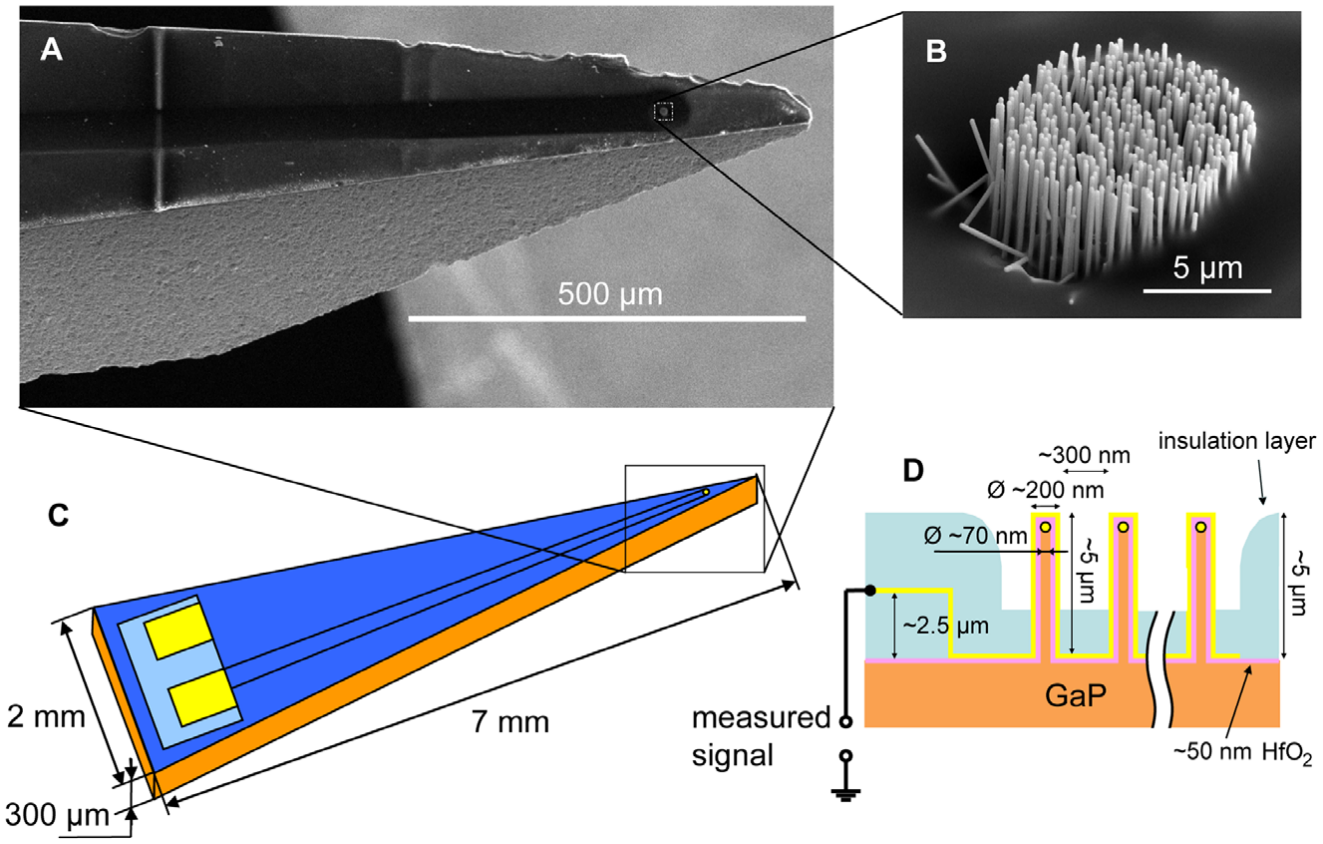
Nanowire-based electrode for acute *in vivo* neuronal signal recordings. (A) Scanning electron microscope (SEM) image of the nanowire-based electrode tip. (B) SEM image of the nanowire-based sensing region made with an array of freestanding vertical gallium phosphide nanowires covered with hafnium oxide and metal film. (C) Layout for the nanowire-based electrode. (D) Schematic for the nanowire geometry and the electrode layered structure.

The nanowire-based probe developed is shown in Figure 1. The sensing part of the probe was based on an array of vertical GaP nanowires regularly spaced on a 12 µm diameter round area with a 500 nm pitch size. The diameter and length of each GaP nanowire were 70 nm (with very little tapering) and 5 µm, respectively. An Au metal film deposited on top of the nanowires constituted the sensing part of the electrode. The effective surface area for the nanowire-based electrode was ∼100 µm^2^. The GaP nanowires and GaP substrate were covered by a 50 nm thick layer of hafnium oxide (HfO_2_) in order to improve the mechanical properties of the nanowires but also to provide an electrical insulation between the GaP material and the metal film, deposited on top of the nanowires. The electrical insulation between the film lead (the metal thin film lead connecting the sensing part with the bonding pad) and the GaP substrate on one side (the bottom insulation) and between the film lead and liquid on the other side (the top insulation) was provided by a 2.7 µm thick polymer coating. Note that only the metal film on top of the nanowire protrusions contributes to the signal measurements since the metal film in between the nanowires is insulated with a polymer film. This probe configuration was chosen in order to ensure that the neuronal recordings were done only with the nanowire-based protrusions, as a proof of concept of nanowire-based electrodes operation *in vivo*. Note that the sensing site, *i.e.* the nanowire array, is located in a shallow groove on one side of the probe. Note also that all the nanowires in the array are electrically connected together.

### Electrical Properties

**Figure 2:**
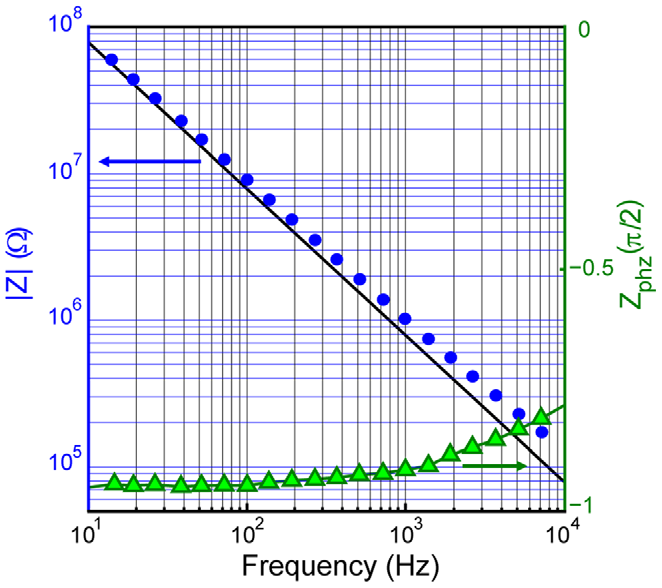
Nanowire-based electrode impedance measured as a function of frequency in 0.9% w/v NaCl water solution. The blue and green dots represent the impedance magnitude and the phase respectively. The black line shows an impedance magnitude calculated for a 200 pF capacitor. The electrode impedance dependence on frequency indicates that the nanowire-based electrodes are mainly coupled to the ionic current in saline solution through an electrolytic capacitor and corresponds to ∼100 µm^2^ Au surface area in contact with liquid.

The electrode impedance, as measured with impedance spectroscopy, was found to be 1.2±0.4 MΩ at 1 kHz. The typical impedance measured at different frequencies is shown in Figure 2. The electrode impedance dependence on frequency indicated that the nanowire-based electrodes were mainly coupled to the ionic current in saline solution through an electrolytic capacitor of approximately 200 pF, corresponding to approximately a 100 µm^2^ Au area in contact with liquid, see black line in Figure 2 [37].

### Acute *In Vivo* Measurements

**Figure 3:**
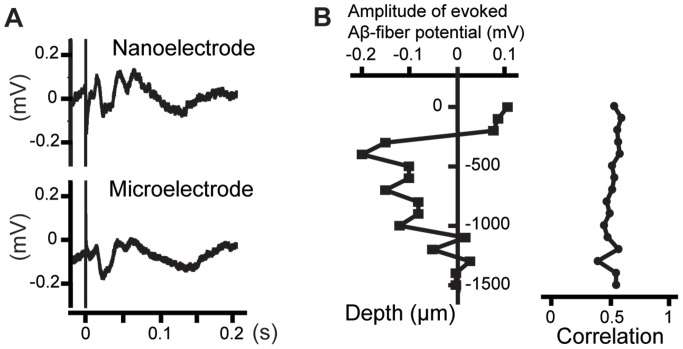
Electrically evoked intracortical field potentials recorded in the rat primary somatosensory cortex (acute measurements). (A) Simultaneous recordings using a nanowire-based electrode and a microwire electrode glued together and implanted 400 µm below the cortex surface (averaged over 32 sweeps). (B) Depth profile of evoked Aβ-fiber potential (filled boxes) recorded by the nanowire-based electrode (plotted for each depth as the peak-valley amplitude, with an onset latency between 10 ms and 20 ms after the stimulation) and correlation coefficients (filled circles) calculated for measurements performed simultaneously with the nanowire-based electrode and the microwire (calculated for the measured data sets of time interval up to 0.43 ms after the stimulation). The measurements show that the neuronal signal is primarily recorded with the nanowire-based sensing part and that the nanowire-based electrode provides acute *in vivo* recordings that are comparable to conventional microelectrodes.

To verify that acute recordings of neuronal activity can be achieved with the nanowire-based sensing part of the electrode probe, measurements were made at different depths in the primary somatosensory cortex of anesthetized rats (Fig. 3). It is known that the field potential evoked by electrical stimulation of the skin has a distinct depth distribution (*cf.* Schouenborg and Kalliomäki [38]). Hence, if the measurements are picked up from the sensor part of the nanowire-based probe, a characteristic depth profile of the field potentials should be obtained.

Figure 3 shows simultaneous acute recordings done with the nanowire-based electrode and with a 33 µm diameter tungsten microwire electrode insulated except for the tip. The two electrodes were glued together to ensure that the sensing sites of respective electrode were inserted to the same cortical depth. Figure 3A shows traces of the evoked field potentials obtained 400 µm below the cortex surface. As can be seen in Figure 3B, the depth profile of the recorded Aβ-fiber evoked field potential was similar for the two electrodes, indicating that both electrodes recorded from the same cortical depth. Moreover, similar traces were obtained by the two electrodes (correlation coefficient 0.60 for traces from same cortical depth shown in Figure 3A).

**Figure 4:**
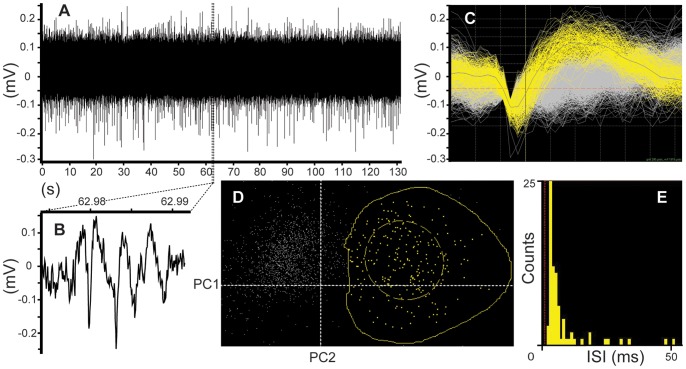
Spontaneous neuron activity recorded with a nanowire-based electrode in the rat primary somatosensory cortex (acute measurements). (A) Raw data with 1811 spikes detected; (B) zoomed region with a neuronal burst; (C) isolated single unit from the recordings in (A), the grid size in x-direction is 0.2 ms; (D) spike cluster view in the principle component space. The cluster corresponds to an isolated single unit as presented in (C). The dashed yellow ellipse in (D) represents the standard deviation for the cluster along the principal component axes and the outer yellow border includes all 213 neural spikes in the cluster. The neuronal unit sorting is based on cluster recognition in principle component space. Here PC1 and PC2 stand for the first and second principle components. (E) The autocorrelation histogram for spike events within the unit, the bin size is 3 ms. The inter spike interval (ISI) for the spike sorting was set to 1.5 ms and resulted in 0.0% spike interference ratio.

In order to further verify that the sensing site of the nanowire-based probe can be used to record localized neuronal activity, acute recordings from spontaneously active cortical neurons were made (Figure 4). The recordings were made at a cortical depth of approximately 1 mm. An isolated single unit activity is shown in Figure 4C. The spike cluster in principle component space for the isolated single unit can be seen in Figure 4D and includes 213 spikes (out of 1811 spikes detected). The neuronal sorting was based on cluster recognition in principle component space [39]. The inter spike interval (ISI) for the spike sorting was set to 1.5 ms and resulted in 0.0% spike interference ratio. The corresponding autocorrelation histogram for the spike events within the unit is presented in Figure 4E.

### Mechanical Stability of the Nanowire-based Probe

**Figure 5:**
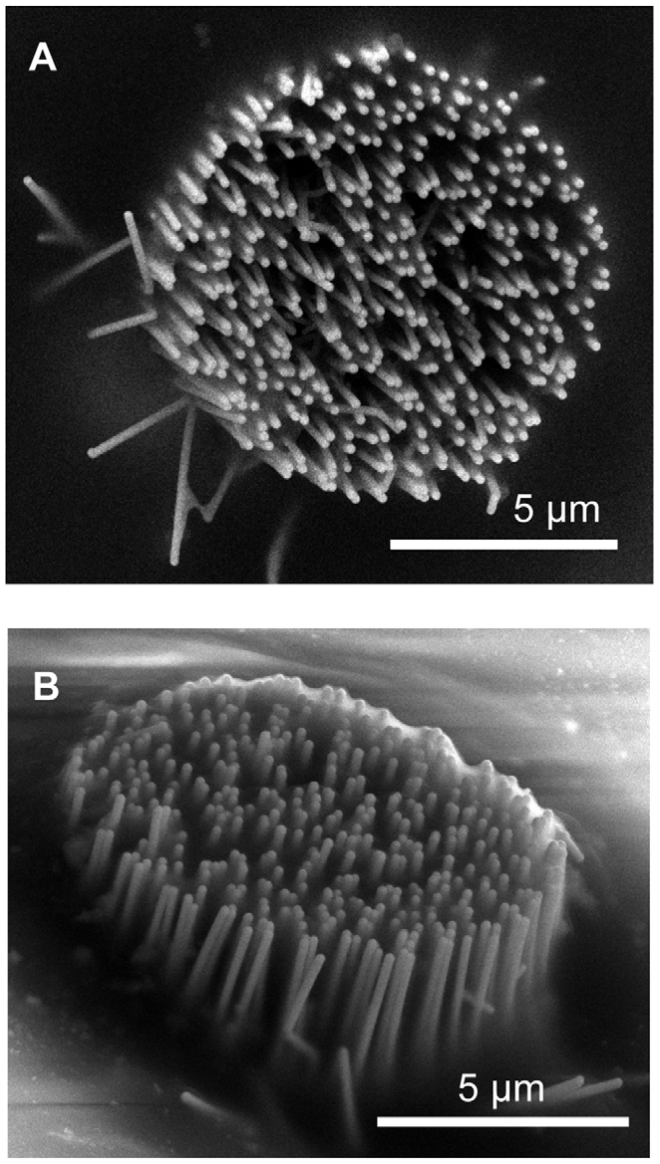
SEM images of the nanowires modified sensing site. The site image presented after a single implantation (A) and after multiple implantations (B) into rat cortex. The same nanowires-based electrode before any implantation can be seen in Figure 1B. Some tissue deposition on the probe after multiple implantations can be seen in Figure 5B.

To test whether the mechanical stability of the nanowire-based sensing part is sufficient to withstand implantations into the brain, multiple implantations and subsequent SEM images were made. Figure 5 shows SEM images of the nanowires sensing site after one (Figure 5A) and after three (Figure 5B) implantations into the rat cortex. For comparison, the same electrode before any implantations is shown in Figure 1B. It can be seen that the nanowire array geometry remains unaffected after multiple brain implantations, showing a remarkable mechanical stability of the nanowires.

## Discussion

A number of microelectrode constructs for chronic recordings have been designed since the start of the neural interface field. The most well-known are the Utah array electrode [40], the Michigan electrode and its later versions [41], [42], the tetrode [43], the cone electrode [44] and plain wire constructions [45]. While these microelectrode constructions have permitted the recordings of numerous neurons in awake animals, the quality of the recordings typically deteriorates over time. Moreover, these implants cause a significant and long lasting tissue reaction, thus causing local tissue reorganization [46]. A common notion is that the glial reaction to the implant contributes to the deterioration of electrode performance, such as reduced signal to noise ratio and loss of unit recordings [7]. However, it is presently not clear to what extent the physical properties, such as size and 3D structure, of the active electrode surface influence the long term performance and glial reactions. In the present study we have taken the first step towards developing a nanostructured electrode that can serve as a model system for an analysis of the long term impact of nanostructured surfaces on neural tissue.

We describe the design, fabrication and functionality of the first generation of GaP nanowire-based electrode with a controlled 3D nanomorphology as a proof of principle that nanowire-based electrodes can provide acute *in vivo* recordings and withstand multiple rat pia mater and nerve tissue penetrations.

We used multiple nanowires electrically connected together in order to reduce the electrode impedance. The effective area for the nanowire-based electrode was approximately 100 µm, which is similar to conventional microelectrodes, and resulted in comparable electrode impedances with linear frequency dependence in logarithmic scale [37], [47]–[49]. The electrode impedance dependence on the frequency indicates that the nanowire-based electrodes are mainly coupled to the ionic current in saline solution through an electrolytic capacitor formed on the metal surface in contact with liquid. It should be noted, that the primary aim of the present study was to test that the nanowire-based electrodes were able to detect neuronal signals. For this reason, we insulated the metal surface between the nanowires in order to ensure that the signal was recorded by the nanowire protrusions only. In the future experiments, the entire area supporting the nanowires can be used for electrical measurements, which would result in a larger electrode area and consequently an improved signal to noise ratio. The surface area of the electrode can also be increased further, by either changing the geometry of the nanowire array, or by modifying the electrode surface with *e.g.* Poly(3,4-ethylenedioxythiophene) (PEDOT) [18]. In the later case the nanowire high aspect ratio geometry may provide a better adhesion for the deposited material as compared to a planar surface.

The simultaneous recordings of evoked intracortical field potentials using the nanowire-based electrode and the microwire electrode are similar and correspond to the standard field potential depth profile, which presents a collective neuronal response to a strong sensory stimulus and has maximum negativity at 0.5–1 mm depth in this cortex area, where the thalamocortical fibers terminate [38]. These results and the spontaneous cortical neuron activity recorded using the nanowire-based electrode prove that the recordings are done primarily with the nanowires modified sensing part.

Importantly, nanowires for electrode surface modification purpose may offer several potential advantages compared to other surface enhanced electrodes: (i) From a biocompatibility point of view, it has been shown *in vitro* that most cellular functions were not affected by nanowire substrates and that the mechanical properties of free-standing nanowire substrates could mimic the natural cell environment [19]–[21]. Recently we showed that even in a worst case scenario, if the nanowires detach from the substrate, the nanowires should not present any substantial risk for the organism [50]. (ii) Nanowires were recently shown to provide a noninvasive neuron pinning [35] and therefore may provide a reliable tissue anchoring, which is important for long-term stable neurophysiological measurements *in vivo*. (iii) The metal film deposited on top of the nanowire structures provides a stable coating and this nano-structured topography has a better mechanical stability compared to other nano-modified electrodes, such as the ones coated with platinum black which is very brittle.

It should be stressed that here we present a first step of the development of a new electrode with several potential advantages. The present electrode design will need further development in order to enable its utility as a model system for analysis of the functional properties of implanted nanostructured neuronal interfaces for chronic use. Most importantly, the size of the GaP substrate used for the nanowire growth needs to be minimized in order to reduce the tissue damage and consequent tissue reaction following implantation. This is a prerequisite for a detailed analysis of the long term influence of the nanotopography, such as pattern, density and length of nanowires, on the electrode performance and tissue reactions. In the future, it may be also interesting to apply different chemical modifications to the electrode surface for the electrode-cell interaction improvement [12], [22] and substitute the gold surface with other metals like platinum or tungsten.

We have previously shown that the nanowires can withstand an implantation in a 1% agar gel, a commonly used *in vitro* model for the mechanical properties of the brain [51]. Here we show that the nanowire-based electrode can indeed withstand multiple pia matter and brain implantations, while staying mechanically intact and providing acute neurophysiological recordings. In the future, embedding the nanowire electrode tip in a dissolvable matrix as recently demonstrated [52], may lessen the tissue damage during the electrode implantation and at the same time protect the electrode against tissue deposition, which is likely to increase the recording quality.

In summary, as a first step towards developing a useful nanostructured device for neurophysiological measurements *in vivo*, a new type of nanowire-based electrode for neuronal signal recordings has been developed, characterized and tested in saline solution and in the rat brain. These measurements present the first functional testing of nanowire-based electrodes in the brain and prove the feasibility of nanowire based electrodes for acute *in vivo* neurophysiological measurements. We believe that, after reduction of the size of the GaP substrate, the nanowire-based electrodes can be used as a model system for studies on the properties of nanomodified neuronal interfaces *in vivo*, since the nanowire geometry and spatial patterning can be controlled with a very high precision.

## Materials and Methods

### Ethics Statement

All procedures were approved in advance by the Malmö/Lund Animal Ethics Committee on Animal Experiments (Permit Number: M120-09). All surgery was performed under anesthesia, and all efforts were made to minimize suffering.

### Nanowire-based Electrode

Arrays of vertical freestanding gallium phosphide (GaP) nanowires were epitaxially grown on the (111)B GaP surface of 300 µm thick double side polished GaP substrates. Substrates of 15×15 mm^2^ were used for the nanowire growth and the electrode processing. The nanowires were grown using metalorganic vapor phase epitaxy (MOVPE) from gold catalytic particles defined with electron beam lithography (EBL).

The GaP substrates were patterned with gold catalytic particles as follows: the EBL resist polymethylmethacrylate (PMMA) A5 950 kDa was spun at 5000 rpm on the sample for 30 s. Subsequently the sample was baked on a hot plate at 160°C for 15 min. Arrays of single pixel dots were defined in the resist using Raith 150 EBL system operating at 20 kV with single-pixel dose of 22 fAs. The samples were developed in a mixture methyl isobutyl cathone/isopropanol (1∶3) for 60 s before rinsing in isopropanol (IPA) for 30 s and blowing dry with nitrogen. Thermal evaporation of a 20 nm thick gold film was done with custom built AVAC thermal evaporator at a base pressure of <10^−6^ mbar. This was followed with a lift-off in acetone at 60°C, rinsing in IPA and blowing dry with nitrogen.

All samples were stored in a nitrogen-filled glovebox until the nanowire growth. The nanowires were grown by MOVPE (AIX200/4, Aixtron AG) from the EBL defined gold particles. Before the growth, the samples were annealed for 10 min at 470°C in the presence of hydrogen and phosphine (H_2_ and PH_3_) in the MOVPE reactor. The nanowire growth was done by supplying trimethylgallium (Ga(CH_3_)_3_) and phosphine at 470°C (precursor molar fractions 10^−5^ and 10^−2^ for trimethylgallium and phosphine respectively) in a hydrogen carrier gas flow of 6 L/min under low pressure (10 kPa). The growth time of few minutes defined the nanowire length of 5 µm. The exact growth time was affected by the growth chamber history and had to be calibrated each time before the growth. The nanowire diameter was determined by the gold particle size and was typically 70 nm. The resulting GaP nanowires grew in the [111] B direction, perpendicular to the surface with little tapering. In this way a high degree of control over the nanowire array geometry (nanowire position, length, and diameter) was achieved.

The nanowires were grown in arrays located on a ∼12 µm diameter circular area with a 500 nm pitch. In order to improve the mechanical properties of the nanowires we deposited a 50 nm thick layer of HfO_2_ using atomic layer deposition (Savannah-100 system, Cambridge NanoTech).

The electrical insulation between the film lead and the GaP substrate (the bottom insulation) was made by spin coating the substrates with 2.7 µm layer of the photosensitive Microposit S1818 (Shipley Company) polymer. A selective removal of the polymer layer from the nanowire sites was accomplished using ultraviolet lithography (UVL): polymer soft baking (hot plate, 115°C, 90 s) was followed by optical exposure (350 s exposure, Mask aligner MJB 4 DUV, Karl Süss MicroTec AG) and development for 90 s in Microposit MF 319 (Shipley Company) developer. The polymer was then hard baked (oven, 200°C, 1 h) and its surface was activated with oxygen plasma (1 min, 5 mbar oxygen pressure, Plasma-Preen System II 862 from Plasmatic System Inc.). A 15 nm Ti layer and 75 nm Au layer were subsequently deposited on the substrates using a magnetron sputter (Orion 5, AJA International). Since the nanowire walls were perpendicular to the substrate, this resulted in approximately 5 nm thick Ti and 25 nm thick Au layers deposited on the nanowire walls, resulting in a final nanowire diameter between 200 and 240 nm.

A positive 5 µm thick Microposit S1818 photosensitive polymer layer was deposited by spin coating on the metal and patterned with UVL to delineate the electrical connections to the nanowire-based recording sites. The excessive metal was subsequently etched through the polymer mask with an Au etch (10 g KI, 2.5 g I_2_, 100 ml H_2_O, 30 s) and Ti etch (5% v/v HF water solution, 10 s) followed by resist removal with Microposit Remover 1165 (80°C, 15 min).

The electrical insulation between the film lead and liquid (the top insulation) was done by spin coating another photoactive 2.7 µm thick Microposit S1818 polymer layer. The underneath hard baked resist surface was activated using oxygen plasma treatment (15 s, 5 mbar) before the spin coating. This resist layer was soft baked and patterned with UVL in the same way as the bottom insulation polymer layer (*vide supra*). This ensured that the metal film on the top part of nanowires and the metal film on the bonding pads were revealed while the connecting film lead was encapsulated. An additional oxygen plasma treatment (60 s, 5 mbar) was performed in order to clean the metal electrode surface from polymer residues. Finally, the electrodes were hard baked at 200°C for one hour.

After the thin film processing the substrates were diced with a sand saw into 7 mm by 2 mm triangular probes. The probes were connected to the external circuitry by 125 µm diameter silver wires using the conducting epoxy EPO-TEK EE129-4 (Epoxy technology Inc.). The wires were isolated from any contact with the liquid using glass capillaries (Harvard Apparatus LTD) to which the probes were fixated with insulating biocompatible epoxy EPO-TEK GE116 (Epoxy technology Inc.). The probe tips were grinded with a specially designed grinding setup, which enabled probe tip trimming without any damage or contamination on the nanowire-based electrode. The grinding setup was made from a standard grinding setup (used for *e.g.* manufacturing micropipette probes for neurophysiological measurements). It was equipped with a tissue wipe for cleaning the grinded GaP material and placed under a microscope for precise probe orientation with respect to the grinding wheel. The trimming facilitated the electrode brain implantation.

### Nanowire-based Electrode Characterization

The nanowire-based electrodes were inspected using SEM and characterized using impedance spectroscopy (potentiostat Reference 600, Gamry Instruments) in a 0.9% NaCl water (saline) solution in a three-electrode arrangement (platinum wire as a counter electrode and Ag/AgCl 3.5 M KCl as a reference electrode) at 22°C over 10 Hz –10 kHz frequency range with 10 mV rms input signal amplitude.

### Acute *In Vivo* Measurements

Female Sprague Dawley rats (Taconic, Denmark) weighing 300–320 g were used for the measurements. The animals were anaesthetized with intraperitoneal injections of ketamine (Ketalar 50 mg/ml, Pfizer, Täby, Sweden), xylazine (Rompun 20 mg/ml, Bayer, Göteborg, Sweden), and attached to a stereotactic frame (KOPF Instruments, USA). Craniotomies were made and the dura mater was removed over the primary somatosensory cortex target area (2–3 cm^2^). More details on the animal handling and anesthetic procedures can be found elsewhere in literature [53].

**Figure 6:**
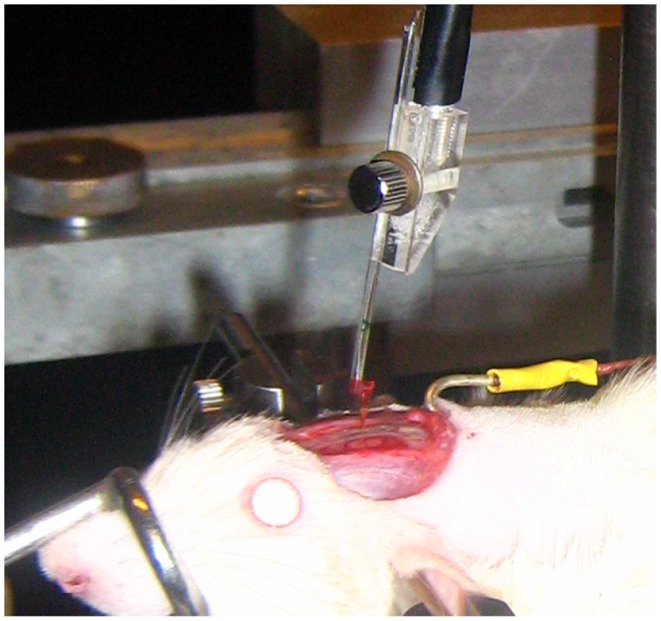
Photograph of the implantation experimental set-up. The nanowire-based electrode is electrically connected to a preamplifier and fixated on a micromanipulator for implantation into the rat cortex. The yellow wire to the right was used as an animal ground.

The experimental set-up for the implantation of the nanowire-based electrodes is shown in Figure 6. The probes were attached to a hydraulic micromanipulator (KOPF Instruments, USA) and electrically connected to a preamplifier. A separate wire (yellow) immersed in the brain liquid was used as the ground electrode. The implantations were made under visual inspection using a microscope at a speed of 1–10 µm s^−1^ to a maximum depth of approximately 2 mm. Acute neurophysiological measurements were done with a preamplifier NL102G, amplification and filtering units of Neuro Log system from Digitimer Ltd. A hardware signal filtering using a notch 5–50 Hz filter was done for the data presented. The data acquisition and processing was done using Spike2 and Signal programs from Cambridge Electronic Design Ltd.

In our experiments, the local field potentials were evoked by an electrical stimulation of the plantar side of the rat hind paw (1.5 mA, 0.5 ms pulses at 1 Hz) and the curves presented were averaged over 32 sweeps. The Aβ-fiber evoked potentials were plotted for each depth as the peak-valley amplitude, with an onset latency between 10 ms and 20 ms after the stimulation. The nanowire-based electrode acute recordings of the evoked potentials were compared to recordings by a 33 µm diameter tungsten wire with formvar insulation (California Fine Wire Company, USA) [41]. The correlation was calculated for the measured data sets of time interval up to 0.43 ms after the stimulation. One correlation coefficient was calculated for each depth. The single unit sorting was done using Offline Sorter (Plexon Inc.) with a user-defined template.
